# The Effect of Polymeric Nanoparticles on Biocompatibility of Carrier Red Blood Cells

**DOI:** 10.1371/journal.pone.0152074

**Published:** 2016-03-22

**Authors:** Daniel Pan, Omayra Vargas-Morales, Blaine Zern, Aaron C. Anselmo, Vivek Gupta, Michael Zakrewsky, Samir Mitragotri, Vladimir Muzykantov

**Affiliations:** 1 Department of Pharmacology and Center for Translational Targeted Therapeutics and Nanomedicine, Perelman School of Medicine, University of Pennsylvania, Philadelphia, Pennsylvania, United States of America; 2 Department of Chemical Engineering and Center for Bioengineering, University of California Santa Barbara, Santa Barbara, California, United States of America; James Cook University, AUSTRALIA

## Abstract

Red blood cells (RBCs) can be used for vascular delivery of encapsulated or surface-bound drugs and carriers. Coupling to RBC prolongs circulation of nanoparticles (NP, 200 nm spheres, a conventional model of polymeric drug delivery carrier) enabling their transfer to the pulmonary vasculature without provoking overt RBC elimination. However, little is known about more subtle and potentially harmful effects of drugs and drug carriers on RBCs. Here we devised high-throughput *in vitro* assays to determine the sensitivity of loaded RBCs to osmotic stress and other damaging insults that they may encounter *in vivo* (*e*.*g*. mechanical, oxidative and complement insults). Sensitivity of these tests is inversely proportional to RBC concentration in suspension and our results suggest that mouse RBCs are more sensitive to damaging factors than human RBCs. Loading RBCs by NP at 1:50 ratio did not affect RBCs, while 10–50 fold higher NP load accentuated RBC damage by mechanical, osmotic and oxidative stress. This extensive loading of RBC by NP also leads to RBCs agglutination in buffer; however, addition of albumin diminished this effect. These results provide a template for analyses of the effects of diverse cargoes loaded on carrier RBCs and indicate that: i) RBCs can tolerate carriage of NP at doses providing loading of millions of nanoparticles per microliter of blood; ii) tests using protein-free buffers and mouse RBCs may overestimate adversity that may be encountered in humans.

## Introduction

Red blood cells (RBCs, bi-concave discoid cells filled with hemoglobin and lacking organelles including the nucleus) represent the most abundant cellular constituent of the blood (>99%) and play an important role in drug delivery. Drug delivery systems circulating in blood encounter RBCs, which may lead to unintended effects of drug on RBCs, and vice versa. Interaction of drug delivery systems (e.g., polymeric nanocarriers) with RBCs and its consequences have a high significance, both mechanistic and translational [1–12].

As carriers, RBCs offer a multitude of advantages including bioavailability, biocompatibility and longevity in circulation (~45 and ~120 days in mice and humans, respectively), which makes them a highly attractive vehicle for vascular delivery of drugs and nanocarriers. Recently, several groups reported interesting characteristics of behavior *in vitro* and in animal models of nanoparticles (NP) non-covalently attached to RBC surfaces [3,13–16]. To summarize these exciting studies, RBC-coupled nanoparticles showed prolonged circulation in mice, reduced uptake in clearance organs (*e*.*g*. liver and spleen), and enhanced targeting and delivery to the lungs, enabled by the transient transfer of nanoparticles from carrier RBCs to the endothelium.

Coupling nanocarriers to the surfaces of mouse RBCs at 50:1 ratio, as was done in these previous studies, did not induce negative effects in RBCs *in vitro* or alter their overall performance *in vivo*. However, little is known about the effect that attached nanocarriers have on RBCs, either as a result of intentional RBC carriage, such as in the above examples, or from unintentional interactions. In this study, we designed a set of high-throughput *in vitro* assays characterizing sensitivity of nanocarrier-carrying RBCs to biologically relevant insults often encountered in circulation; specifically osmotic, mechanical, oxidative and complement stress, as well as RBC agglutination. These tests can provide a pre-screening for selection of formulations exhibiting the least sensitivity to these biological insults, and likely enhanced performance, prior to *in vivo* testing in both preclinical and clinical settings.

## Materials and Methods

### Ethics Statement

All animal studies were were carried out in strict accordance with Guide for the Care and Use of Laboratory Animals as adopted by National Institute of Health, approved by University of Pennsylvania IACUC under protocol 805013. Mice were anesthetized with ketamine/xylazine/acepromazine. Mice were bled at one time point, alternating eyes to bleed from. At the end, all animals were euthanized by cervical dislocation and confirmed by detection of no heart beat.

All studies involving human subjects were approved by the University of Pennsylvania Institution Review Board under protocol 822534. Written informed consent from donors was obtained for the use of blood samples in this study. Blood samples were destroyed after the study. Names and any personal information about individual participants were not taken.

### Blood collection

CJ7BL/6J male mice were purchased from the Jackson Laboratory (Bar Harbor, ME). All mice were housed in a temperature and humidity controlled environment (18–23°C with 40–60% humidity under a 12-hour light-dark cycle) with ad libitum access to food (Labdiet 5010 autoclavable rodent diet, Brentwood, MO) and water.

Blood donation of human voluntary donors took place at the University of Pennsylvania. A volume of 4mL of whole blood was collected in either a vial containing ~ 3.2% Na citrate (BD Vacutainer) or Na heparin 75 USP Units (BD Vacutainer). Blood collected in Na Heparin was used to avoid calcium depletion for complement assays. In addition, a volume of 4mL of whole blood was collected in serum collecting tubes (BD Vacutainer). Blood from CJ7BL/6J mice, which also took place at the University of Pennsylvania, was collected in 20% Na heparin. Both human and murine blood was spun at 1000g for 10 min at 4°C to isolate plasma or serum. Serum was stored at 4°C for 3h until use. Plasma was discarded. A buffy layer containing white blood cells, observed in the interface between plasma and erythrocytes, was also removed and discarded.

Isolated erythrocytes (RBCs) were washed by adding ice cold 1x Dulbecco’s-Phosphate-Buffered-Saline (DPBS) pH 7.4 up to 12mL total volume and pipetting gently up and down to mix buffer with RBC extensively. RBC suspension was centrifuged again (451g, 15 min, 4°C) and supernatant was discarded. This wash step was repeated for a total of 3 times.

### Attachment of Nanoparticles to RBCs

Briefly, 200nm carboxylated polystyrene particles (Polysciences) were washed in water, centrifuged at 15,000g for 20 min and later suspended in 4% sodium citrate pH 7.4. NPs were incubated with either murine or human RBCs at a ratio of 50:1; 200:1; or 2000:1 for 1 h under constant rotation at 4°C. NP-RBC solution was washed with ice cold DPBS three times at 100g for 8 min to remove unattached NPs.

### RBC Osmotic Fragility

Osmotic fragility assay was performed on freshly obtained erythrocytes in the presence of an anticoagulant. RBCs and RBC-NP suspensions were washed three times with ice cold DPBS and re-suspended in ice cold DPBS at 10% hematocrit, followed by incubation with different ratios of NPs to RBCs for 60 min at 4°C. RBCs were washed three times with ice cold 4% DPBS to wash off unbound NPs. RBC lysis was monitored by the release of hemoglobin upon incubation of cells with salt concentrations ranging from 0mM to 150mM for ~ 0 min (e.g. immediately removed from salt solution) at 37°C. RBC suspensions were centrifuged at 13,400g for 4 min and the absorbance of the supernatant was recorded at 540nm by TECAN Infinite M200 plate reader. Each sample in water was taken as 100% RBC lysis. All RBC suspensions in the following fragility assays followed this same procedure.

### RBC Mechanical Fragility

Mechanical fragility assay was performed on freshly obtained erythrocytes in the presence of an anticoagulant. All erythrocytes samples were subjected simultaneously to the same mechanical stress. RBC and RBC-NP suspensions of 1.0% hematocrit along with 8x4mm glass beads (Pyrex) in DPBS were rotated 360° for different time periods at 24 rpm at 37°C. The control samples were not subjected to the glass beads. The hemoglobin released from the RBCs during rotation was immediately assayed, as was the free hemoglobin in the control samples. Free hemoglobin was transferred into a new tube and was centrifuged at 13,400g for 4 min.

### RBC Oxidative Fragility

Hydrogen peroxide-induced lysis was performed on freshly obtained erythrocytes in the presence of an anticoagulant. All erythrocytes samples were subjected simultaneously to the same oxidative stress. RBC and RBC-NP suspensions of 1.0% hematocrit with the addition of 3mM H_2_O_2_ in DPBS were rotated 360° for different time periods at 24 rpm at 37°C. The control samples were not subjected to H_2_O_2_. The hemoglobin released from the RBCs during rotation was immediately assayed, as was the free hemoglobin in the control samples.

### RBC Complement Lysis

Complement lysis assay was performed as previously described [17–20]. Streptavidin (SA) and 6-biotinylaminocaproic acid N-hydroxysuccinmide ester (long arm biotin ester, BxNHS) were purchased from Sigma. Briefly, fresh isolated erythrocytes from heparinized blood were washed with three times with cold DPBS before the addition of biotin. BxNHS, dissolved in DMF, was added to RBC suspension of 10% hematocrit and incubated at room temperature for 30 minutes. Biotinylated (B) RBCs were then washed four times with cold DPBS. Streptavidin (1mg/mL in DPBS) was then added to 10% biotinylated RBC and later incubated at room temperature for 30 min. Unbound streptavidin was removed by centrifugation. RBC, RBC-NP, and RBC-B-SA suspensions of 1.0% hematocrit with the addition of the complement obtained from fresh homologous serum, diluted ½ in DPBS containing Ca^2+^ and Mg^2+^ were rotated 360° for 4h at 24 rpm at 37°C. The addition of biotin-streptavidin (B-SA) to RBC suspension was used as a control. The hemoglobin released from the RBCs during rotation was immediately assayed for lysis, as was the free hemoglobin in the control samples. Each sample in water was taken as 100% RBC lysis.

### RBC Agglutination assay

Agglutination assay was performed on freshly obtained erythrocytes in the presence of an anticoagulant. RBC and RBC-NP suspensions of 1.0% hematocrit were dispensed onto a 96 well U-shaped plates. The results were visually accessed for agglutination after 1 hour at 37°C after the RBC suspension adsorbed had fully sedimented. RBC suspension in dog serum was used as a positive control of agglutination process. To confirm RBC agglutination, RBC and RBC-NP suspensions were observed using a 25x objective lenses on a Micromaster microscope (Fisher Scientific) equipped with micro-camera.

## Results

### Characterization of the test system effects of RBC hematocrit and animal species

Naïve RBCs were first investigated alone, without attachment of nanocarriers, so as to establish a baseline with regards to RBC sensitivity and resistance against a variety of biological insults. RBC concentration (% hematocrit) was shown to be reversely proportional to hemolysis during an osmotic fragility test. At intermediate levels of osmotic stress, in this case caused by hypotonic buffer, the percent of hemolysis increased as RBC concentration decreased **(S1 Fig)**. This finding agrees well with previous studies [17]. To avoid very low readings of optical density, typical of highly diluted RBC samples, 1% RBC suspensions were used throughout the study. Additionally, mouse RBCs are dramatically more sensitive than human RBCs to osmotic, mechanical and oxidative stresses/insults (**S2 Fig)**, which highlights the need to be cautious when correlating potential human efficacy in mouse models that, in some cases, are less durable.

### Sensitivity of NP-RBC to osmotic stress

**Fig 1:**
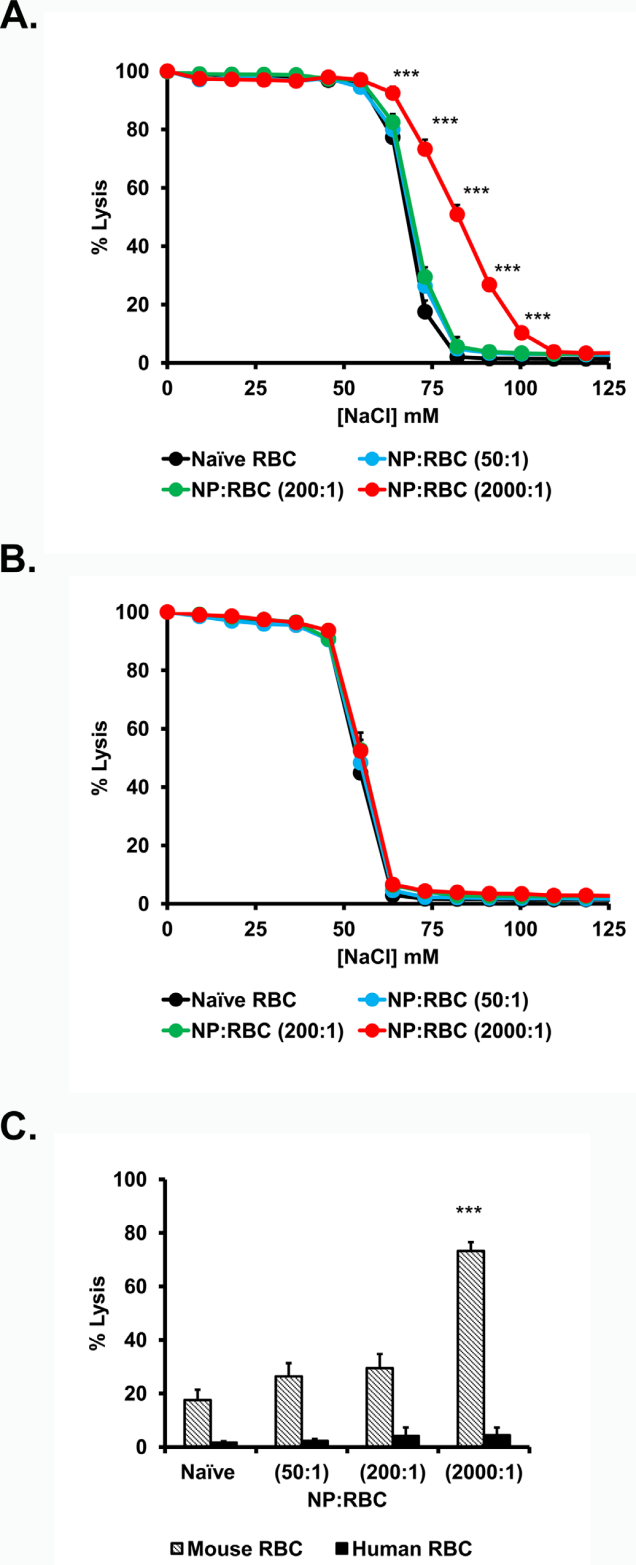
Osmotic fragility of red blood cells with 200nm nanoparticles adsorbed onto their surface at 1.0% hematocrit. Hemolytic curves for mouse (A) and human (B) NP:RBCs were obtained after immediate exposure to different [NaCl]. Curves were determined for NP:RBC ratio (50:1, 200:1, and 2000:1). Percent hemolysis of different RBC/NP:RBC ratios at 73mM NaCl (C). Values are means (n = 4–6) ± SD. (***, P<0.001 vs naïve RBC). Please note that some deviation bars are too small to be evident.

Attachment of NP to RBCs at NP/RBC ratios 50:1 and 200:1 did not alter hemolysis of mouse and human RBCs in sub-physiological osmotic conditions (e.g. 73mM NaCl), whereas NP loading at NP/RBC ratio of 2000:1 aggravated osmotic lysis of mouse but not human RBCs (**Fig 1**). Osmotic lysis occurred rapidly, in these cases the percentage of RBC hemolysis does not increase significantly from ~0 min to 3h of both mouse and human RBCs within tested range of NP:RBC ratios.

### Sensitivity of RBC-NPs to low level shear stress

RBC resistance to mechanical stress is one of the key physiological features that contribute to RBC longevity in the bloodstream. Hemodynamic stress can cause mechanical damage to RBCs, resulting in rupture of the cell’s membrane which leads to hemolysis [21–24]. To investigate how NP-carriage effects RBC resistance to mechanical stress, we first tested RBC sensitivity to a relatively prolonged low level shear stress (rotation at 24rpm at 37°C for 46h), similar to what RBCs repetitively encounter in the microcirculation.

**Fig 2:**
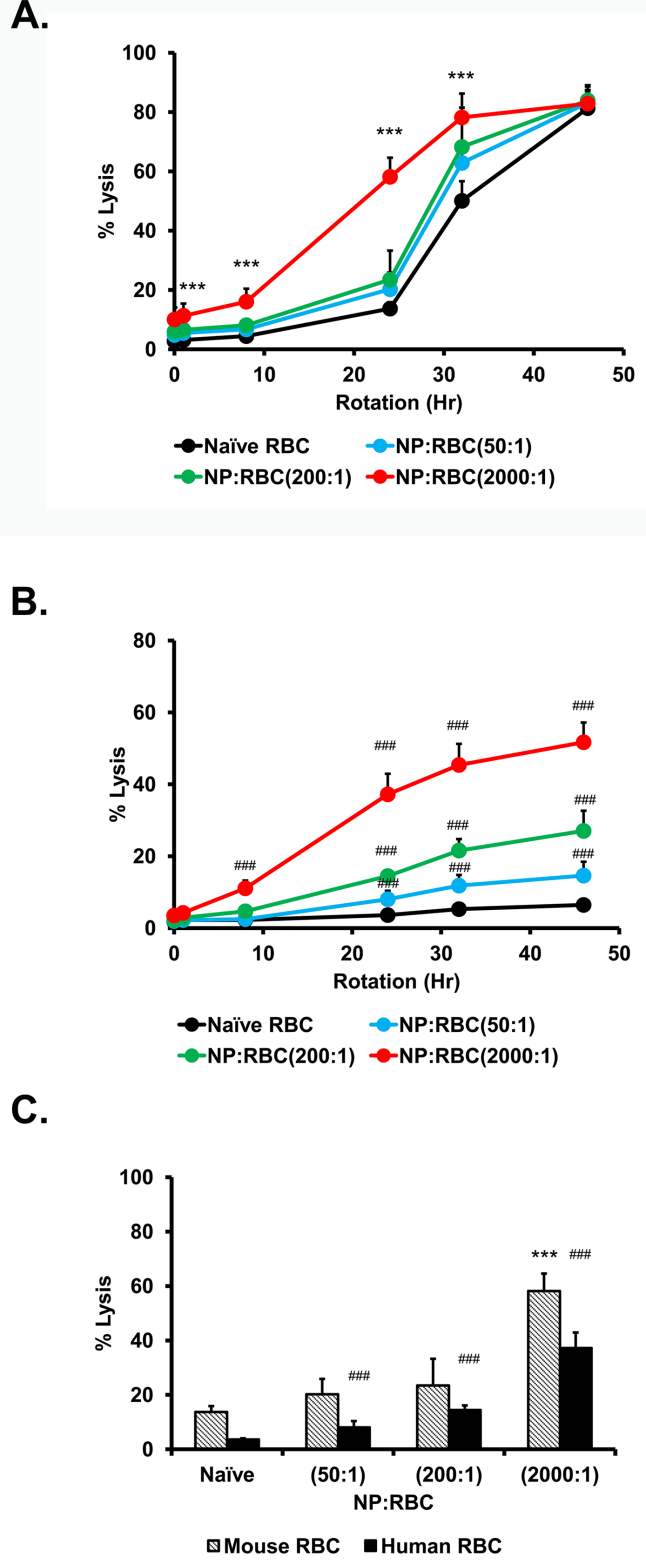
Effect of low stress over time on red blood cells with 200nm nanoparticles adsorbed onto their surface at 1.0% hematocrit. Hemolytic curves for mouse (A) and (B) human RBC/NP:RBCs were obtained after constant rotation at 24 rpm at 37°C for up to 46h. Curves were determined for NP:RBC ratio (50:1; 200:1; 2000:1). Percent hemolysis of different NP:RBC ratios at 24h (C). Values are means (n = 5) ± SD. (***, P<0.001 vs naïve mouse RBC) (###,P<0.001 vs naïve human RBC).

As shown in **Fig 2A**, naive mouse RBCs showed low levels of hemolysis at 8h (3% lysis); increased rotation times resulted in an increase in hemolysis from 15% after 24h to 80% at 46h. Within the time interval at which mouse RBCs withstand mechanical stress (8 hours and partially 24 h), NP loading to RBC at ratios of 50:1 and 200:1 did not sensitize RBCs. Loading at NP/RBC ratio of 2000:1 markedly aggravated the hemolysis induced by mechanical stress (15% lysis after 8h; 60% lysis after 24h). However, as was the case with osmotic insults, human RBCs were also more resistant to mechanical stresses for all NP/RBC ratios. As shown in **Fig 2B**, NP loading to human RBCs at 200:1 ratio slightly aggravated hemolysis, whereas loading at 2000:1 ratio markedly aggravated RBC damage caused by mechanical stress.

### Sensitivity of RBC-NP to vigorous mechanical insult

**Fig 3:**
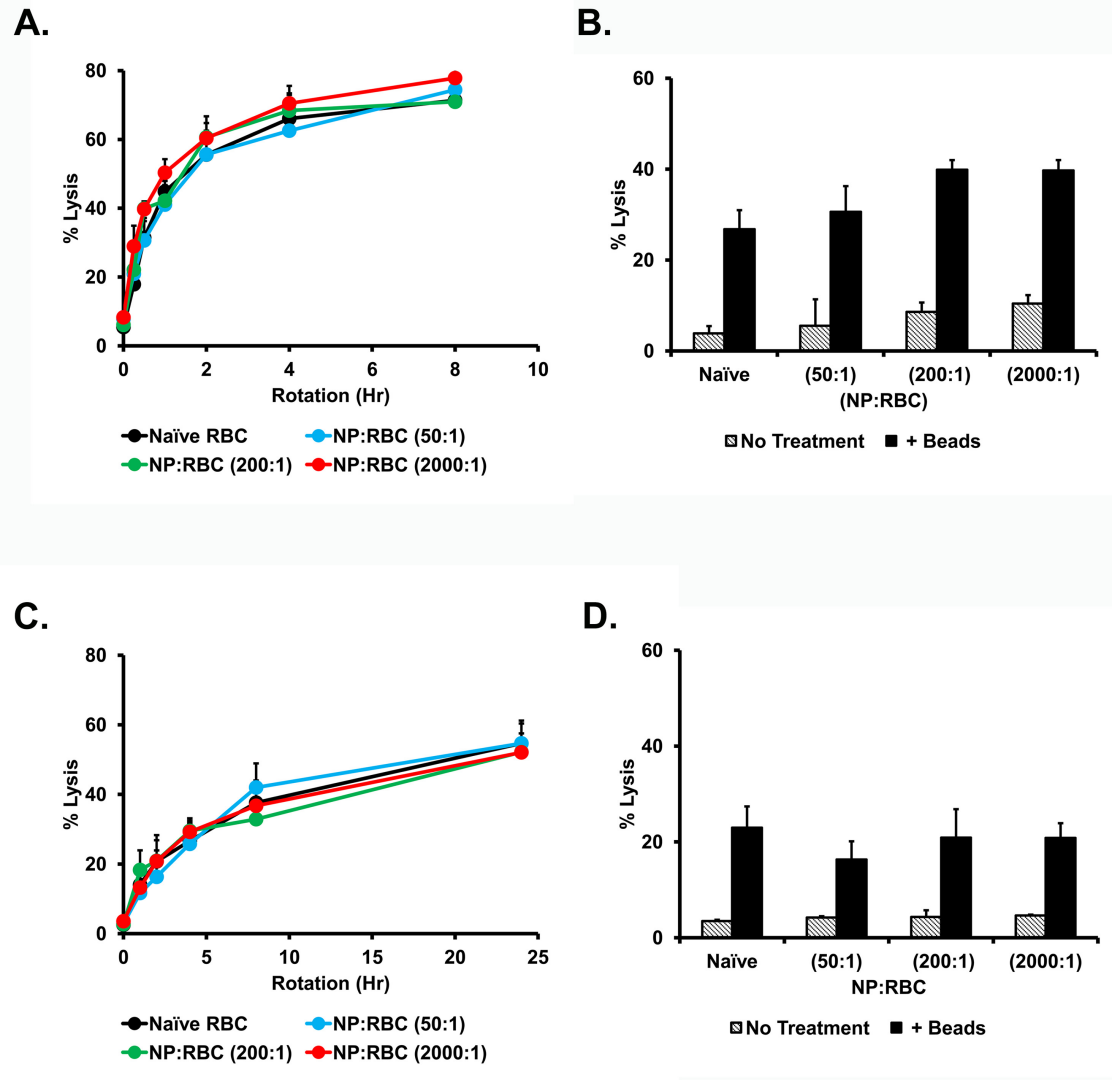
Mechanical fragility of red blood cells with 200nm nanoparticles adsorbed onto their surface at 1.0% hematocrit. Percent hemolysis of mouse NP:RBCs (A) and human NP:RBCs (C) (50:1; 200:1; and 2000:1) were obtained after constant rotation with glass beads at 24 rpm at 37°C for up to 8h and 24h, respectively. Percent hemolysis of different NP:RBC ratios for mouse RBC at 0.5h (B) and human RBC at 2h (D). Values are means (n = 4–5) ± SD. Please note that some deviation bars are too small to be evident.

In the heart chambers and aorta, RBCs encounter excessively high levels of shear stress from the damaging effects of vigorous collisions with the valves and vascular walls. To model severe mechanical stress, glass beads were added to the RBC suspension and additionally subjected to the rotational shear stress. Even short (15 min) incubation under these conditions caused ~20% and ~5% hemolysis of naive mouse and human RBCs, respectively (**S2 Fig**). This hemolysis was modestly increased for RBC-NPs after 30 min exposure for mouse RBCs at high levels of NP load (**Fig 3A & 3B**), however, this was not the case for human RBCs even at the highest NP load for 2 hours of exposure (**Fig 3C & 3D**).

### Sensitivity of RBC-NPs to oxidative insult combined with low level shear stress

Damage to RBCs *in vivo* may be enhanced under pathological conditions. For example, oxidative stress is a common component of many human maladies including inflammation and ischemia-reperfusion, where H_2_O_2_ is one of the key reactive oxygen species (ROS) released from activated phagocytes in inflammation and ischemia-reperfusion. RBC exposure to ROS is likely to occur in the microvasculature, where mild hemodynamic conditions permit accumulation of leukocytes and ROS.

**Fig 4:**
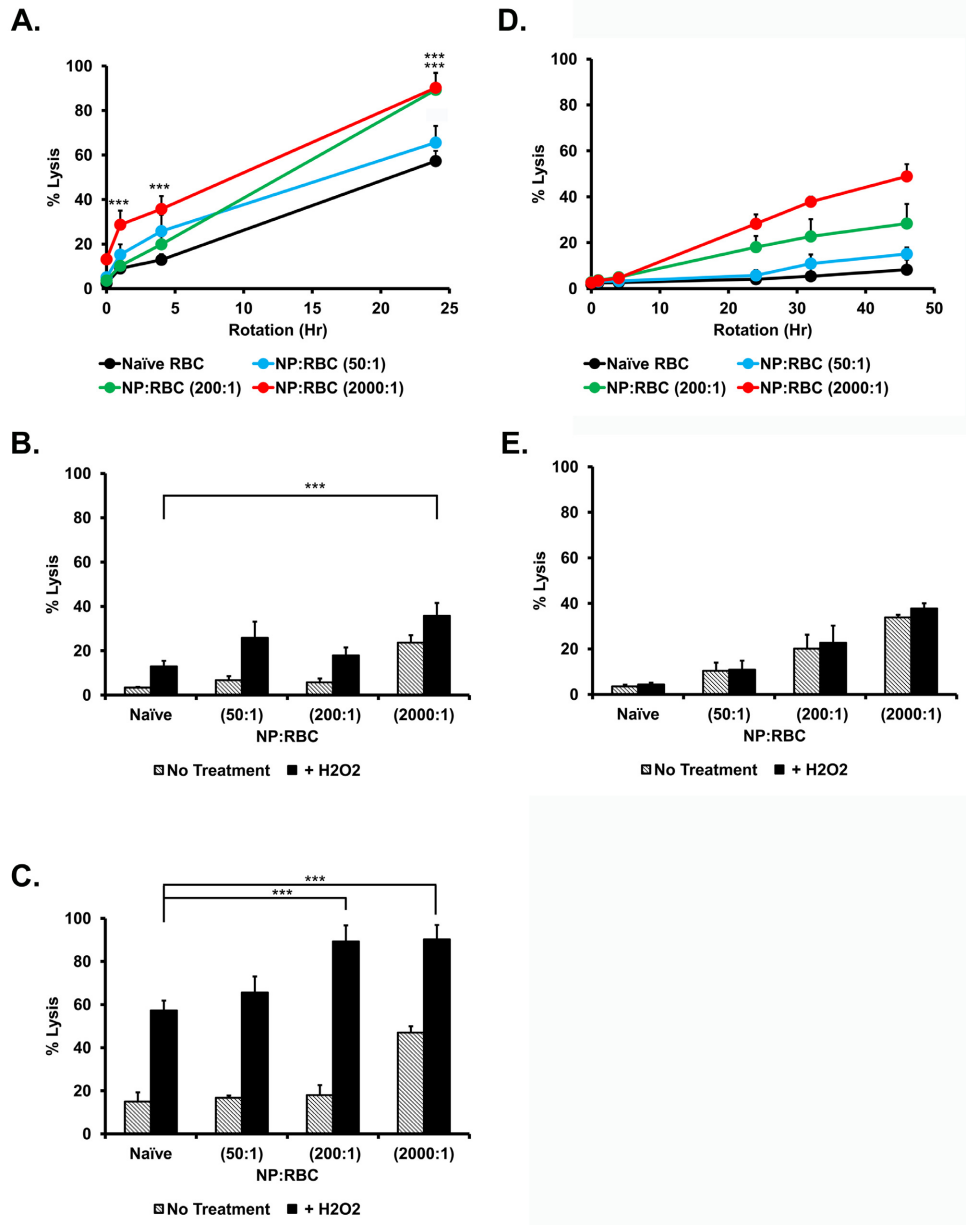
Oxidative fragility of red blood cells with 200nm nanoparticles adsorbed onto their surface at 1.0% hematocrit. Percent hemolysis of mouse (A,B,C) and human (D,E) RBC/NP:RBCs (50:1; 200:1; and 2000:1) were obtained after rotating at 24 rpm at 37°C for up to 24h and 46h, respectively, after being challenged with 3mM H_2_O_2_. Percent hemolysis of different NP:RBC ratios for mouse RBC at 4h (B), 24h (C) and human RBC at 32h (E). Values are means (n = 4–5) ± SD. (***,P<0.001 vs naïve RBC). Please note in (D) lysis is caused by adsorption of NPs, not by H_2_O_2_. See Fig 2B for controls without H_2_O_2_.

Accordingly, we assessed the sensitivity of RBCs and NP/RBCs exposed to 3mM H_2_O_2_ at low level of shear stress. The results (**Fig 4A–4C**) show that at these conditions ROS aggravates hemolysis of mouse RBC in a time-dependent manner and NP loading at any tested ratio, including NP/RBC 50:1. It should be noted that manifestation of H_2_O_2_ aggravation requires several hours and becomes especially pronounced after 24 hours. As such, since human RBCs have continued to outperform mouse RBCs in regards to insult resistance, and given that microvasculature is the important common site of oxidative stress (see "Discussion"), longer-timepoints were investigated and perhaps not surprisingly, the attachment of NPs to human RBCs at any ratio did not enhance oxidative hemolysis (**Fig 4D and 4E**).

### Sensitivity of NP/RBCs to complement lysis under low level shear stress

Complement is the key component of the innate defense system that recognizes, destroys and marks microorganisms and altered (e.g., damaged) host cells for phagocytosis. Senescent and modified RBCs are sensitive to complement lysis and do not have membrane-repairing enzymes employed by nucleated cells. We examined whether the attachment of NPs onto RBCs increases the RBC susceptibility to complement-mediated lysis via alternative pathway that relies on direct, antibody-bypassing attack of predisposed RBCs [18].

**Fig 5:**
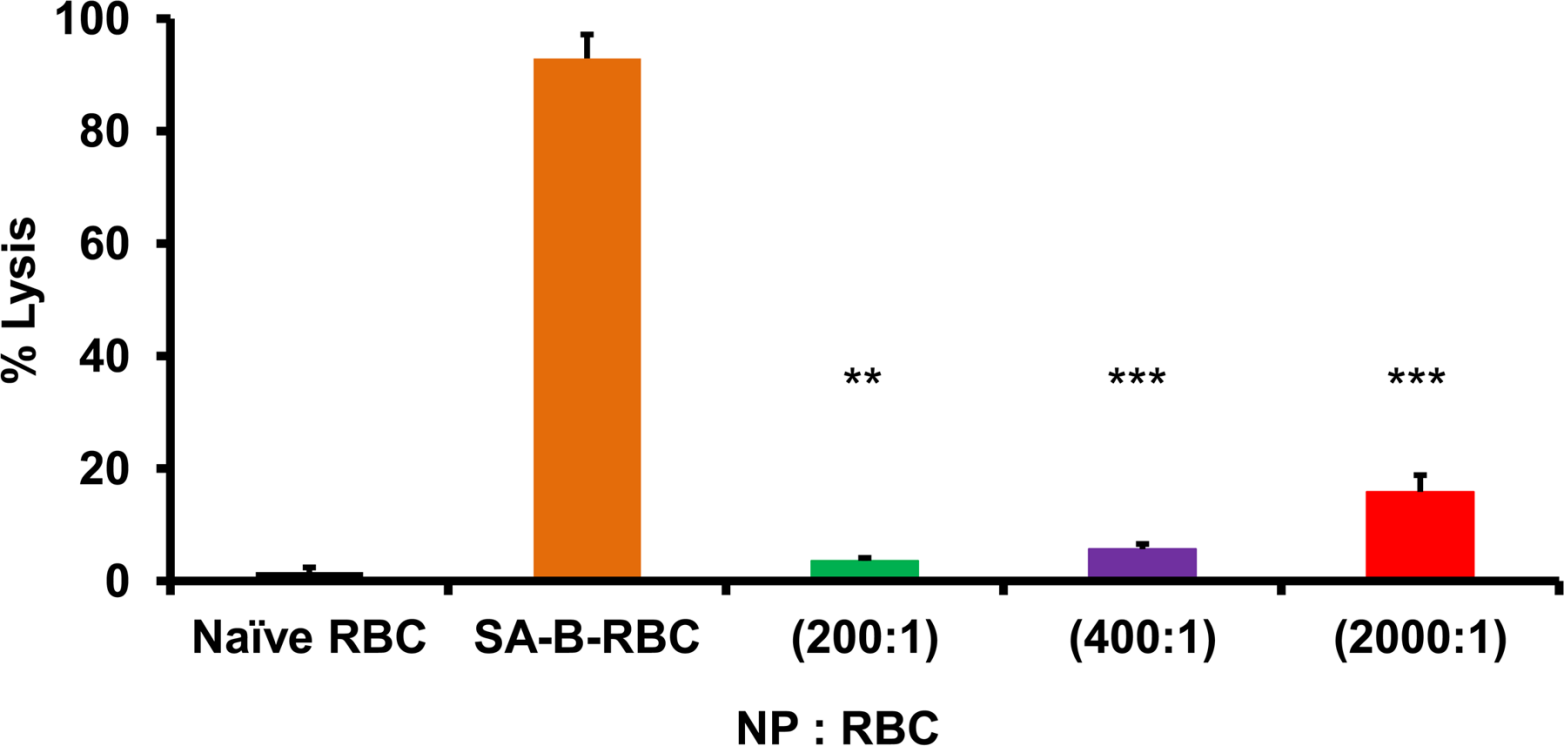
Complement lysis of human RBCs with 200nm nanoparticles adsorbed onto their surface at 1.0% hematocrit. Hemolytic curves for human RBC/NP:RBCs were obtained after the addition of complement obtain from serum rotating at 37°C for 4h. The addition of streptavidin-biotin (SA-B) was used as a control. Curves were determined for NP:RBC ratio (200:1; 400:1; 2000:1). Values are means (n = 4) ± SD. (**, P<0.01 ***, P<0.001 vs. naïve RBC). Please note that some deviation bars are too small to be evident.

For the positive control, we attached streptavidin to biotinylated RBC which leads to RBC hemolysis via the alternative complement pathway due to inactivation of the RBC membrane glycoproteins DAF and CD59, which normally control homologous complement [18–20, 25–29]. Here, human RBCs were exclusively investigated given their enhanced insult resistance. Human RBCs were not lysed at any concentration of analogous serum, whereas streptavidin attachment to biotinylated RBC led to nearly complete hemolysis in the analogous serum (**Fig 5**). NP loading at NP/RBC ratios of 200:1 and 400:1 did not aggravate complement lysis further. However, at NP/RBC ratio of 2000:1, nearly a 3-fold increase of lysis was detected.

### Sensitivity of NP/RBCs to agglutination

To further evaluate other possible effects of NP on RBCs, agglutination processes were analyzed. This phenomenon reflects abnormal changes in RBC plasticity, adhesiveness and propensity to absorb plasma agglutinins, all of which occur in a number of pathologies. For example, the formation of rouleaux, where RBCs appear as stacked coins and individual RBCs cannot be distinguished (aggregates), are associated with hematological and other diseases.

**Fig 6:**
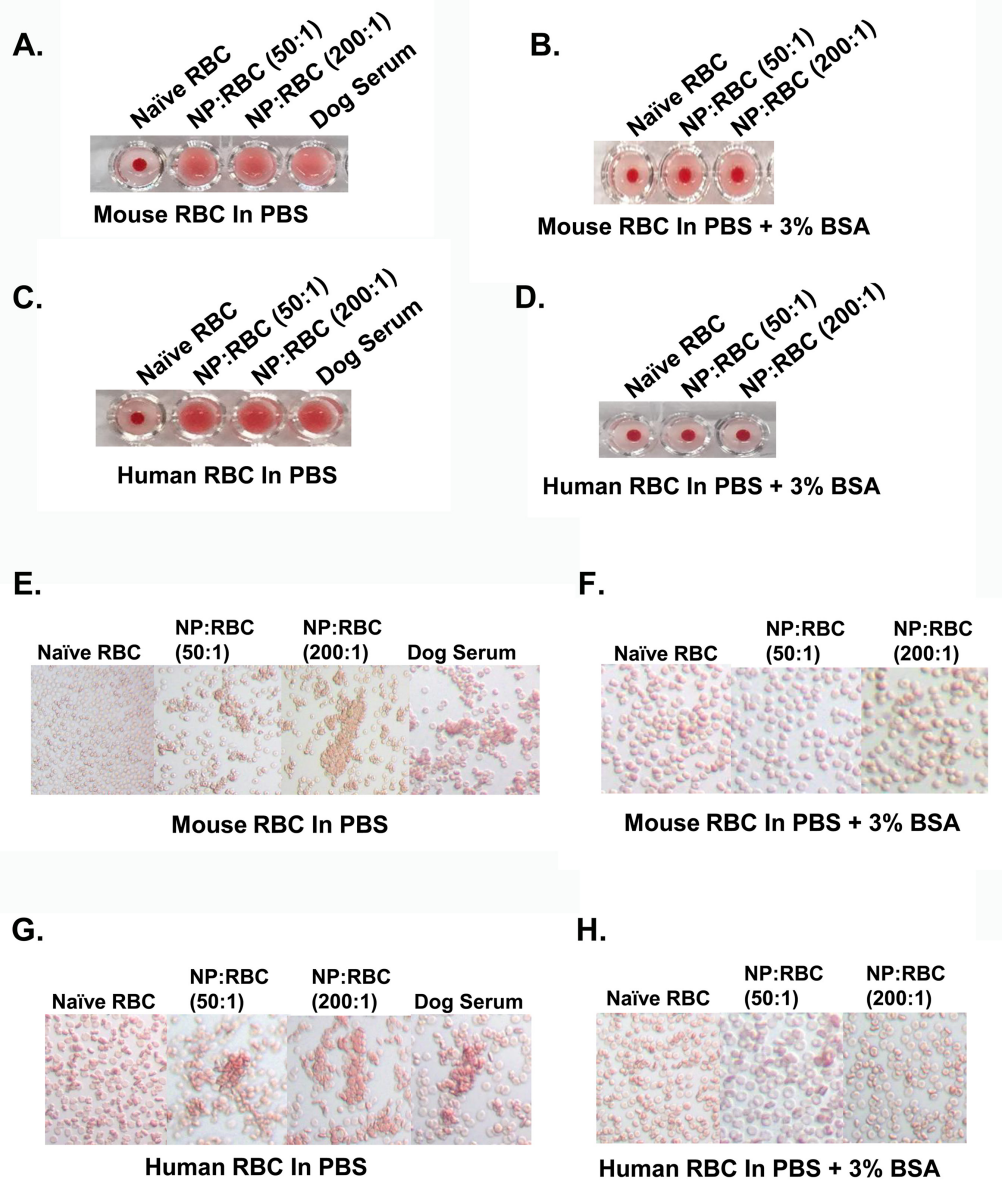
Agglutination of red blood cells with 200nm nanoparticles adsorbed onto their surface at 1.0% and 10% hematocrit in buffer with and without BSA. Agglutination was visualized on a U-shaped bottom plate for mouse (A,B) and human (C,D) NP:RBCs (50:1 and 200:1). The deposition of RBC on the bottom as a dot indicates a negative agglutination reaction. Microscopic imaging of RBC agglutination. Agglutination was also visualized with light microscope at 250x magnification for both mouse (E,F) and human (G,H) at 10% Hematocrit. Dog serum served as a positive control. Data shown are representative of three independent experiments.

A standard dilution assay in a V-shape titration micro-plate revealed that NP loading on RBCs even at low NP/RBC ratio of 50:1 led to detectable RBCs agglutination in buffer (**Fig 6A and 6C**). The agglutination induced by the NPs was confirmed by microscopy showing large clumps of NP-loaded, but not naive RBCs (dog serum was used as a positive control of RBC agglutination) (**Fig 6E and 6G**). However, agglutination process was inhibited in the presence of BSA, even at a NP/RBC ratio as high as 200:1 (**Fig 6B and 6D**). No RBC clumps were observed by light microscope (**Fig 6F and 6H**).

## Discussion

Effect on cells and tissues encountered on the route to therapeutic target site is an important factor of any drug delivery system from a performance and safety viewpoint. As such, reliable and sensitive tests are needed for pre-screening of multiple iterations of nanocarriers prior to expensive and low-throughput animal experiments. Red blood cells (RBCs), encountered by all circulating delivery systems offer a relatively high-throughput, reproducible and biologically relevant model system for testing unintended effects of carriers. RBCs are arguably the most available cellular material from any species including humans, which helps to explain expansion of studies characterizing adversity of drug delivery systems using relatively simple readouts of RBC agglutination and hemolysis [30].

On the other hand, RBCs have a potential as drug carriers. In particular, RBCs may help in optimizing nanocarrier circulation, circumventing uptake by the reticuloendothelial system (RES) [1–3, 12, 31]. Other methods, such as coating nanocarriers by PEG, or using elongated and elastic nanocarriers have been shown to decelerate carrier's clearance [32–33]. In theory, this goal can also be accomplished by coupling nanocarriers onto the surface of RBCs, given their ability to naturally circulate for long-times [1–4]. In addition to prolongation of carrier's lifetime in the bloodstream, RBC offers mechanisms for masking attached cargo compounds by the glycocalyx [5–11] and for their delivery to targets accessible from blood—thrombi, immune cells, macrophages, endothelial cells and the RES sinuses, to name a few [2,7,9,34–37].

Recently, several labs embarked on exploration of RBC-mediated carriage of nanocarriers [3–4,13, 38]. In particular, a non-covalent absorption of polymeric nanoparticles (NPs, 200 nm polystyrene spheres used as model drug delivery vehicles) to naive mouse RBC cardinally changed behavior of the resultant NP-RBC complexes injected in the bloodstream of mice. Somewhat expected, circulation time of RBC-bound NP was remarkably longer than that of free NP. Importantly, coupling of NP to RBC at ratios up to 50:1 did not accelerate elimination of the carrier RBC. Further, RBC carriage enabled transfer of reversibly associated NP to the pulmonary vasculature [3]. These results imply potential utility of RBCs as a "super-carrier" optimizing the PK and endothelial delivery of synthetic carriers, notwithstanding that the NP used in that study, rigid non-degradable polymeric spheres lacking PEG coating, represent the prototype model that undoubtedly will be surpassed by more advanced iterations with fully biocompatible features.

Both testing biocompatibility of diverse drug delivery systems and using RBCs themselves as drug carriers require sensitive high-throughput *in vitro* tests for detecting potentially harmful changes in RBCs. In this work we describe a set of relatively simple assays based on detection of RBC hemolysis and agglutination that allow analysis of multiple samples, dilutions and conditions imitating distinct factors that occur in circulation and may provoke or aggravate RBC damage, destruction and elimination.

This aspect of RBC drug delivery and modifications needs a rigorous analysis, both *in vivo* and *in vitro*. Of course, no *in vitro* model can fully address the complex conditions and influences experienced by RBCs in circulation. However, *in vitro* testing is more ethically coherent, economical, provides high-throughput analysis of numerous conditions and allows studies of human RBCs. In this context, the present study provides a template of functional tests of specific insults that may be encountered by RBCs in circulation.

Assays employing RBCs from healthy animals and subjects might underestimate potential damaging or sensitizing effects of the cargo attached to or encapsulated in RBC. Presumably, RBC-mediated carriage of drugs and nanocarriers will eventually be employed in sick patients, not healthy individuals. Pathological mechanisms may both affect RBCs predisposing them to damage, or/and lead to production of additional damaging factors and agents. For example, oxidative stress is a prevalent pathological pathway in a plethora of human maladies. Therefore, there is a substantial probability of NP-RBC encountering an additional insult of oxidative stress including reactions with membrane lipids and proteins which produce lipid peroxidation and alter membrane structure [39–47]. Oxidative stress is involved in number of factors that contribute to RBC aging and removal from circulation [48–49]. However, RBCs possess the antioxidant defense, consisting of non-enzymatic antioxidants including glutathione and enzymatic antioxidants including catalase [50–52] and peroxiredoxin-2 [53–54]. This may help to explain delayed manifestation and relatively modest amplitude of oxidative damage of RBC loaded with NP.

The issue of relevance of *in vitro* assays of sensitivity of the carrier RBCs to conditions *in vivo* is complex. Testing effect of individual damaging factors is mechanistically informative and offers highly controlled high-throughput analyses, but it does not reflect the real situation *in vivo*, where several damaging mechanisms combine in their effect on RBCs in an unpredictable and uncontrolled fashion. On the other hand, *in vitro* assays may overestimate the damage. Using mouse RBCs illustrates this point. In addition, changes caused by isolation, energy starvation and lack of stabilizing effect of normal blood plasma all may additionally sensitize isolated RBCs versus their counterparts circulating in the bloodstream.

Of course, an ideal battery of *in vitro* tests would maximally adequately correspond to situation *in vivo*. However, in the context of design of drug delivery systems, the overestimation of danger is arguably less problematic than an opposite outcome, from the standpoint of the patient safety and early awareness of possible translational problems, prompting design of timely and effective countermeasures.

## Conclusion

RBCs continuously undergo constant exposure to insults during their lifespan, which results in continuous biochemical, physical, and structural changes. These changes impair the ability of RBCs to transport oxygen and eventually trigger their removal from circulation by RES. Contact of RBCs with non-biological objects including nanoparticles, polymers used for masking RBC antigenic determinants, drug delivery systems targeted to RBCs, and any drug delivery system introduced intravenously may adversely alter RBCs and their functions [55–62]. The results of this study indicate that the non-covalent adsorption of model NPs to mouse and human RBCs is not detrimental at ratios of and below NP/RBC 200:1. This result is consistent with data obtained *in vivo*, showing minimal effect of NP on circulation of labeled carrier RBC. We used mouse RBCs and human RBCs for the sake of consistency with our previous studies *in vivo* in mice and gauging potential translational utility of animal data. As compared with mouse counterparts, human RBCs showed higher resistance to all challenges and NP loading doses. This result is encouraging in terms of potential translational aspects: most likely, data obtained using mouse RBCs overestimate potential adversity of RBC modifications.

## Supporting Information

S1 Fig Osmotic fragility of red blood cells at different hematocrits. (A) Hemolytic curves for freshly obtained mouse RBC at different hematocrits from 2% to 0.05% were obtained after immediate exposure to different [NaCl]. (B) % hemolysis at 73mM NaCl (solid filled bars) and at 150mM NaCl (hashed filled bars). Values are means (n = 5) ± SD. Please note that some deviation bars are too small to be evident.
(TIF)

S2 Fig Fragility of red blood cells at 1% hematocrit from different species. **(A).** Osmotic fragility of freshly obtained naïve mouse and human RBC after immediate exposure to different [NaCl]. **(B).** Hemolytic curves for freshly obtained naïve mouse and human RBC under low stress were obtained after constant rotation at 24 rpm at 37°C for up to 46h. **(C)** Mechanical fragility of freshly obtained naïve mouse and human RBC (were obtained with (triangles) and without (circles) glass beads at 24rpm at 37°C for up to 8h and 24h, respectively. **(D)** Oxidative fragility of freshly obtained naïve mouse and human RBC were obtained after rotating at 24rpm at 37°C for up to 24h and 46h, respectively, after being challenged with 3mM H_2_O_2_ (triangles). Negative controls without H_2_O_2_ treatment are also shown (circles). Values are means (n = 4–6) ± SD. Please note that some deviation bars are too small to be evident.
(TIF)

## References

[1] Rossi L, Serafini S, Pierige F, Antonelli A, Cerasi A, Fraternale A, et al Erythrocyte-based Drug Delivery. Expert Opin on Drug Delivery. 2005; 2(2): 311–322. 10.1517/17425247.2.2.311 16296756

[2] Muzykantov V. (2010). Drug Delivery by Red Blood Cells: Vascular Carriers Designed by Mother Nature. Expert Opin on Drug Delivery. 2010; 7(4): 403–427. 10.1517/17425241003610633 PMC2844929 20192900

[3] Anselmo A, Gupta V, Zern B, Pan D, Zakrewsky M, Muzykantov V, et al Delivering nanoparticles to lung while avoiding liver and spleen through adsorption on red blood cells. ACS Nano. 2013; 7(12): 1129–1137. 24182189 10.1021/nn404853z PMC4128963

[4] Anselmo A and Mitragotri S. Cell-mediated delivery of nanoparticles. Taking advantage of circulatory to target nanoparticles. J Control Release. 2014; 190: 531–541. 10.1016/j.jconrel.2014.03.050 24747161 PMC4142097

[5] Murciano J, Medinilla S, Eslin D, Atochina E, Cines D, and Muzykantov V. Prophylactic fibrinolysis through selective dissolution of nascent colts by tPA-carrying erythrocyte. Nat Biotechnol. 2003; 21(8): 891–896. 12845330 10.1038/nbt846

[6] Zaitsev S, Danielyan K, Murciano J, Ganguly K, Krasik T, Taylor R, Pincus S, Jones S, Cines D, and Muzykantov V. Human complement receptor type 1-directed loading of tissue plasminogen activator on circulating erythrocytes for prophylactic fibrinolysis. Blood. 2006; 108(6): 1895–1902. 16735601 10.1182/blood-2005-11-012336 PMC1895545

[7] Zaitsev S, Spitzer D, Murciano J, Ding B, Tliba S, Kowalska M, et al Targeting of a mutant plasminogen activator to circulating red blood cells for prophylactic fibrinolysis. J Pharmacol Exp Ther. 2010; 332(3): 1022–1031. 10.1124/jpet.109.159194 19952305 PMC2835436

[8] Zaitsev S, Spitzer D, Murciano J, Ding B, Tliba S, Kowlaska M, et al Sustained thrombophrophylaxis mediated by an RBC-targeted pro-urokinase zymogren activated at the site of clot formation. Blood. 2010; 115(25): 5241–4248. 10.1182/blood-2010-01-261610 20410503 PMC2892950

[9] Zaitsev S, Kowalska M, Neyman M, Carnemolla R, Tliba S, Ding B. et al Targeting recombinant thrombomodulin fusion proteins to red blood cells provides multifaceted thromboprophylaxis. Blood. 2012; 119(20): 4779–4785. 10.1182/blood-2011-12-398149 22493296 PMC3367878

[10] Danielyan K, Ganguly K, Ding B, Atochin D, Zaitsev S, Muricano J, et al Cerebrovascular thromboprophylaxis in mice by erythrocyte-coupled tissue-type plasminogen activator. Circulation. 2008; 118(14): 1442–1449. 10.1161/CIRCULATIONAHA.107.750257 18794394 PMC2711540

[11] Gersh K, Zaitsev S, Cines D, Muzykantov, and Weisel J. Flow-dependent channel formation in clots by an erythrocyte-bound fibrinolytic agent. Blood. 2011; 117(18): 4964–4967. 10.1182/blood-2010-10-310409 21389322 PMC3100703

[12] Koshkaryev A, Sawant R, Deshpande M and Torchilin V. Immunoconjugates and long circulating systems: origins current state of art and future directions. Advanced Drug Delivery Reviews. 2013; 65(1): 24–35. 10.1016/j.addr.2012.08.009 22964425 PMC3638731

[13] Chambers E and Mitragotri S. Long circulating nanoparticles via adhesion on red blood cells: mechanism and extended circulation. Exp Bio Med. 2007; 233(7): 958–966. 17609513

[14] Anselmo AC, Kumar S, Gupta V, Pearce AM, Ragusa A, Muzykantov V, et al Exploiting shape, cellular-hitchhiking and antibodies to target nanoparticles to lung endothelium: Synergy between physical, chemical, and biological approaches. Biomaterials. 2015; 68:1–8. 10.1016/j.biomaterials.2015.07.043 26241497

[15] Chen LQ, Fang L, Ling J, Ding CZ, Kang B, and Huang CZ. Nanotoxicity of Silver Nanoparticles to Red Blood Cells: Size Dependent Adsorption, Uptake, and Hemolytic Activity. Chem Res Toxicol. 2015; 28(3): 501–509. 10.1021/tx500479m 25602487

[16] Zhao Y, Sun X, Zhang G, Trewyn BG, Slowing II, and Lin VSY. Interaction of Mesoporous Silica Nanoparticles with Human Red Blood Cell Membranes: Size and Surface Effects. ACS Nano. 2011; 5(2): 1366–1375. 10.1021/nn103077k 21294526

[17] Muzykantov V, Samokhin G, Smirnov M, and Domogatsky S. Hemolytic complement activity assay in microtitration plates. J. Appl. Biochem. 1985; 7(3): 223–227. 3902766

[18] Muzykantov V, Smirnov M, and Samokhin G. Avidin acylation prevents the complement-dependent lysis of avidin-carrying erythrocytes. Biochem J. 1991; 273(Part 2): 393–397. 1991038 10.1042/bj2730393 PMC1149858

[19] Muzykantov V, Smirnov M, and Samokhin G. Strepavidin-induced lysis of homologous biotinylated erythrocytes. Evidence against the key role of the avidin charge in complement activation via the alternative pathway. FEBS Letters. 1991; 280(1): 112–114. 2009954 10.1016/0014-5793(91)80216-p

[20] Muzykantov V, Smirnov M, and Klibanov A. Avidin attachment to biotinylated amino groups of the erythrocyte membrane eliminates homologous restriction of both classical and alternative pathways of the complement. FEBS Letters. 1993; 318(2): 1065–1069. 10.1016/0014-5793(93)80002-c 8440366

[21] Kuypers F. Red cell membrane damage J Heart Valve Dis. 1998; 7(4): 387–395. 9697059

[22] Yen J, Chen S, Chern M, and Lu P. The effect of turbulent viscous shear stress on red blood cell hemolysis. J Artif Organs. 2014; 17(2): 178–185. 10.1007/s10047-014-0755-3 24619800

[23] Sallam A and Hwang N. Human red blood cell hemolysis in a turburent shear flow: contribution of Reynolds shear stress. Biorheology. 1984; 21(6): 783–797. 6240286 10.3233/bir-1984-21605

[24] Down L, Papavassiliou D, and O'Hear E. Significance of extensional stresses to red blood cell lysis in a shearing low. Ann Biomed Eng. 2011; 39(6): 1632–1642. 10.1007/s10439-011-0262-0 21298343

[25] Muzykantov V, Seregina N, Smirnov M. Fast lysis by complement and uptake by liver of avidin-carrying biotinylated erythrocytes. Int J Artif Organs. 1992; 15(10): 622–627. 1428212

[26] Muzykantov V, Smirnov M, and Samokhin G. Avidin-induced lysis of biotinylated erythrocytes by homologous complement via the alternative pathway depends on avidin’s ability of multipoint binding with biotinylated membrane. Biochim Biophys Acta. 1992; 1107(1): 119–125. 1616915 10.1016/0005-2736(92)90336-k

[27] Muzykantov V, Smirnov M, and Klibanov A. Avidin attachment to red blood cells via a phospholipid derivative of biotin provides complement-resistant immunoerythrocytes. J Immuno Methods. 1993; 158(2): 183–190. 10.1016/0022-1759(93)90212-p 8429223

[28] Muzykantov V and Murciano J. Attachment of antibody to biotinylated red blood cells: immuno-red blood cells display high affinity to immobilized antigen and normal biodistribution in rats. Biotechnol Appl Biochem. 1996; 24(Part 1): 41–45. 8756393

[29] Muzykantov V, Murciano J, Taylor R, Atochina E, and Herraez A. Regulation of the complement-mediated elimination of red blood cells modified with biotin and streptavidin. Anal Biochem. 1996; 241(1): 109–119. 8921172 10.1006/abio.1996.0384

[30] Evans BC, Nelson CE, Yu SS, Beavers KR, Kim AJ, Li H, et al Ex Vivo Red Blood Cell Hemolysis Assay for the Evaluation of pH-responsive Endosomolytic Agents for Cytosolic Delivery of Biomacromolecular Drugs. J Vis Exp. 2013; 73: e50166 10.3791/50166 23524982 PMC3626231

[31] Villa CH, Pan DC, Zaitsev S, Cines DB, Siegel DL, and Muzykantov VR. Delivery of drugs bound to erythrocytes: new avenues for an old intravascular carrier. Ther Deliv. 2015; 6(7): 795–826. 10.4155/tde.15.34 26228773 PMC4712023

[32] Brazile D, Prud'homme C, Bassoulett M, Marland M, Spenlehauer G, and Veillard M. Stealth Me. PEG-PLA nanoparticles avoid uptake by the mononuclear phagocytes system. J. Pharm Sci. 1996; 84(4): 493–498. 10.1002/jps.2600840420 7629743

[33] Perry J, Reuter K, Kai M, Herlihy K, Jones W, Luft C, et al Napier M, Bear J, and DeSimone J. PEGylated PRINT nanoparticles: the impact of PEG density on protein binding, macrophage association, biodistribution and pharmacokinetics. Nano Lett. 2012; 12(10): 5304–5310. 10.1021/nl302638g 22920324 PMC4157665

[34] Serafini S, Rossi L, Antonelli A, Fraternale A, Cerasi A, Crinelli R, et al Drug Delivery through Phagocytosis of Red Blood Cells. Transfus Med Hemother. 2004; 31(2): 92–101.

[35] Godfrin Y, Horand F, Franco R, Dufour E, Kosenko E, Bax B, et al (2012). International seminar on the red blood cells as vehicles for drugs. Expert Opin Bio Ther. 2012; 12(1): 127–133. 22023703 10.1517/14712598.2012.631909 PMC3492745

[36] Cremel M, Guerin N, Horand F, Banz A, and Godfrin Y. Red blood cells as innovative antigen carrier to induce specific immune tolerance. Int J. Pharm. 2013; 443(1–2): 39–49. 10.1016/j.ijpharm.2012.12.044 23305866

[37] Mukthavaram R, Shi G, Kersari S, and Simberg D. Targeting and depletion of circulating leukocytes and cancer cells by lipophilic antibody-modified erythrocytes. J Control Release 2014; 183: 146–153. 10.1016/j.jconrel.2014.03.038 24685706 PMC4683104

[38] Atukorale P, Yang YS, Bekdemir A, Carney R, Silva P, Watson N, et al Influence of the Glycocalyx and Plasma Membrane Composition on Amphiphilic Cold Nanoparticles Associated with Erythrocytes. Nanoscale. 2015; 7: 11420–11432. 10.1039/c5nr01355k 26077112 PMC6309694

[39] Becker P, Cohen C, and Lux S. (1986). The effect of mild diamide oxidation on the structure and function of human erythrocytes spectrin. J. Bio Chem. 1986; 261(10): 4620–4628. 3957910

[40] Clemens R and Waller H. Lipid peroxidation in erythrocytes. Chem. Phys. Lipids. 1987; 45(2–4): 251–268. 3319229 10.1016/0009-3084(87)90068-5

[41] Wagner G, Chiu D, Qui H, Heath R, and Lubin B. Spectrin oxidation correlates with membrane vesiculation in stored RBCs. Blood. 1987; 69(6):1771–1781. 3580578

[42] Hebbel R, Leung A, and Mohandas N. Oxidation-induced changes in microrheologic properties of the red blood cell membrane. Blood. 1990; 76(5): 1015–1020. 2393710

[43] Bartosz G.Erythrocyte aging: physical and chemical membrane changes: Gerontology. 1991; 37(1–3): 33–67. 2055498 10.1159/000213251

[44] Waugh E, Narla M, Jackson C, Mueller T, Suzuki T, and Dale G. (Rheologic properties of senescent erythrocytes: loss of surface area and volume with red blood cell age: Blood. 1992; 79(5): 1351–1358. 1536958

[45] Gutteridge J. Lipid peroxidation and antioxidants as biomarkers for tissue damage. Clin Chem. 1995; 41(12 part 2): 1819:1828. 7497639

[46] Nagabagu E, Gulyani S, Earley C, Culter R, Mattson M, and Rifkind J. Iron-deficiency anemia enhances red blood cell oxidative stress. Free Radic. Res. 2008; 42(9): 824–829. 10.1080/10715760802459879 19051108 PMC2730642

[47] Barodka V, Nagababu E, Mohanty J, Nyhan D, Berkowitz D, Rifkind J, et al New insights provided by a comparsion of impaired deformability with erythrocyte oxidative stress for sickle cell disease. Blood Cells Mol Dis. 2014; 52(4): 230–235. 10.1016/j.bcmd.2013.10.004 24246527

[48] Mandal D, Baudin-Creuza V, Bhattacharyya A, Pathak S, Delaunay J, Kundu M, et al Caspase 3-mediated proteolysis of the N-terminal cytoplasmic domain of the human erythroid anion exchange 1 (band 3). J. Biol. Chem. 2003; 278(52): 52551–52558. 14570914 10.1074/jbc.M306914200

[49] Grey J, Kodippili G, Simon K, and Low P. Identification of contact sites between ankyrin and band 3 in the human erythrocyte membrane. Biochemistry. 2012; 51(34): 6838–6846. 22861190 10.1021/bi300693k PMC3448790

[50] Gonzalez R, Auclair C, Voisin E, Gautero H, Dhermy D, and Boivin P. Superoxide dismutase, catalase, and glutathione peroxidase in red blood cells from patients with malignant disease. Cancer Res. 1984; 44(9): 4137–4139. 6589047

[51] Muzykantov V, Sakharov D, Domogatsky S, Goncharov N, and Danilov S. Directed targeting of immunoerythrocytes provides local protection of endothelial cells from damage by hydrogen peroxide. Am J Pathol. 1987; 128(2): 276–285. 3618728 PMC1899616

[52] Nagababu E, Monhanty J, Bhamidipaty S, Ostera G, and Rifkind J. Role of the membrane in the formation of heme degradation products in red blood cells. Life Sci. 2010; 86(3–4): 133–138. 10.1016/j.lfs.2009.11.015 19958781 PMC2819203

[53] Lee T, Kim S, Yu S, Kim S, Park D, Moon H, et al Peroxiredoxin II is essential for sustaining life span of erythrocytes in mice. Blood. 2003; 101(12): 5033–5038. 12586629 10.1182/blood-2002-08-2548

[54] Nagababu E, Mohanty J, Friedman J, Rifkind J. Role of peroxiredoxin-2 in protecting RBCs from hydrogen peroxide-induced oxidative stress. Free Radic. Res. 2013; 47(3): 164–171. 10.3109/10715762.2012.756138 23215741 PMC5911927

[55] Scott M, Murad K, Koumpouras F, Talbot M, and Eaton J. Chemical camouflage of antigenic determinants: “Stealth” erythrocytes. Proc Natl Acad Sci USA. 1997; 94(14): 7566–7571. 9207132 10.1073/pnas.94.14.7566 PMC23862

[56] Murad K, Mahany K, Brugnara C, Kuypers F, Eaton J, and Scott M. Structural and functional consequences of antigenic modulation of red blood cells with methoxypoly(ethylene glycol). Blood. 1999; 93(6): 2121–2127. 10068687

[57] Scott M, Murad K, Koumporouras K, Talbot M, and Eaton J. Chemical camouflage of antigenic determinants. Stealth erythrocytes. Proc Natl Acad Sci USA. 1997; 94(14): 7566–7571. 9207132 10.1073/pnas.94.14.7566 PMC23862

[58] Liu Z, Janzen J, and Brooks D. Adsorption of amphilphilic hyperbranched polyglycerol derivates onto red blood cells. Biomaterials. 2010; 31(12): 3364–3373. 10.1016/j.biomaterials.2010.01.021 20122720

[59] Lin J, Hua W, Zhang Y, Li C, Xue W, Yin J, et. al Effect of poly(amidoamine) dendrimers on the structure and activity of immune molecule. Biochim Biophys Acta. 2015; 1850(2): 419–425. 10.1016/j.bbagen.2014.11.016 25463324

[60] Li C, Jin J, Liu J, Xu X, and Yin J. Stimuli-Responsive Polypropylene for the Sustained Delivery of TPGS and Interactions with Erythrocytes. ACS Appl Mater Interfaces. 2014; 6(16): 13956–13967. 10.1021/am503332z 25051204

[61] Gundersen S, and Palmer A. Conjugation of Methoxpolyethylene Glycol to the Surface of Bovine Red Blood Cells. Biotechnol Bioeng. 2007; 96(6): 1199–1210. 17009332 10.1002/bit.21204

[62] Kontos S, Kourtis I, Dane K, and Hubbell J. Engineering antigens for in site erythrocytes binding induces T-cell deletions. Proc Natl Acad Sci USA. 2013; 110(1): E60–68. 10.1073/pnas.1216353110 23248266 PMC3538192

