# Supplementary figures and images for: The Effect of Polymeric Nanoparticles on Biocompatibility of Carrier Red Blood Cells
Source: PLoS One. 2016 Mar 22;11(3):e0152074. doi: 10.1371/journal.pone.0152074 (PMC4803339; doi:10.1371/journal.pone.0152074)

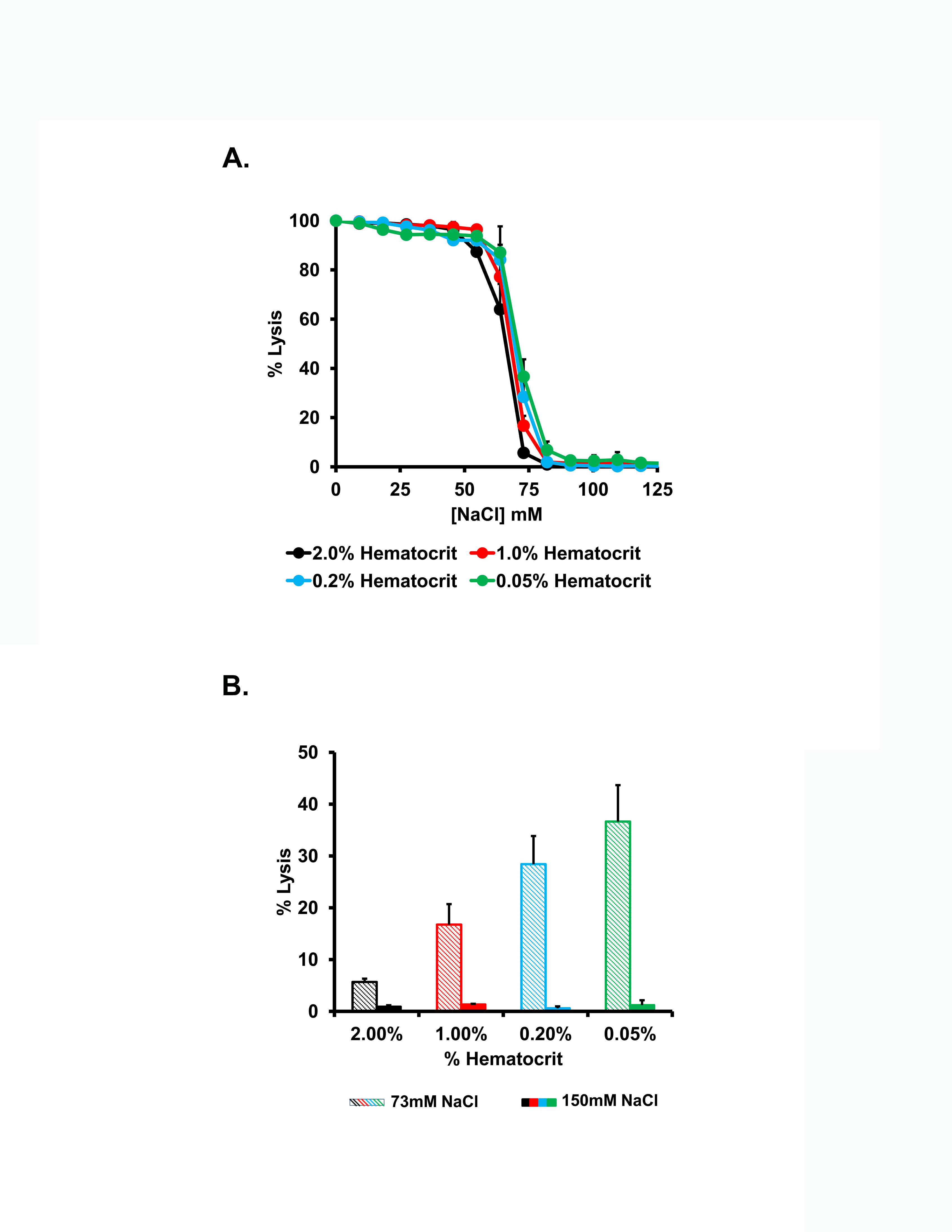

Supplement: S1 Fig — (A) Hemolytic curves for freshly obtained mouse RBC at different hematocrits from 2% to 0.05% were obtained after immediate exposure to different [NaCl]. (B) % hemolysis at 73mM NaCl (solid filled bars) and at 150mM NaCl (hashed filled bars). Values are means (n = 5) ± SD. Please note that some deviation bars are too small to be evident. (TIF) [file pone.0152074.s001.tif]

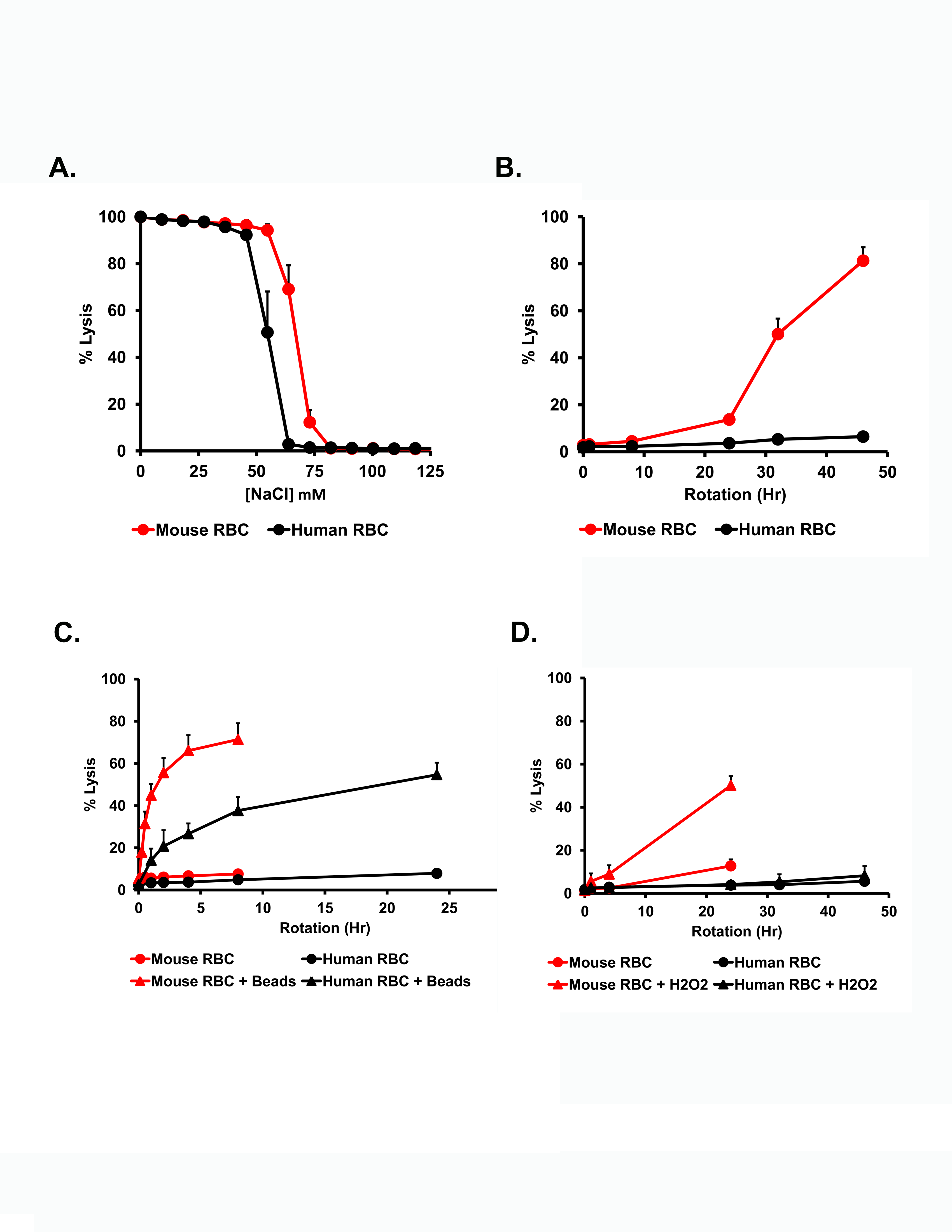

Supplement: S2 Fig — (A). Osmotic fragility of freshly obtained naïve mouse and human RBC after immediate exposure to different [NaCl]. (B). Hemolytic curves for freshly obtained naïve mouse and human RBC under low stress were obtained after constant rotation at 24 rpm at 37°C for up to 46h. (C) Mechanical fragility of freshly obtained naïve mouse and human RBC (were obtained with (triangles) and without (circles) glass beads at 24rpm at 37°C for up to 8h and 24h, respectively. (D) Oxidative fragility of freshly obtained naïve mouse and human RBC were obtained after rotating at 24rpm at 37°C for up to 24h and 46h, respectively, after being challenged with 3mM H2O2 (triangles). Negative controls without H2O2 treatment are also shown (circles). Values are means (n = 4–6) ± SD. Please note that some deviation bars are too small to be evident. (TIF) [file pone.0152074.s002.tif]
